# FDG imaging with long-axial field-of-view PET/CT in patients with high blood glucose—a matched pair analysis

**DOI:** 10.1007/s00259-024-06646-5

**Published:** 2024-02-22

**Authors:** Clemens Mingels, Luis Weissenrieder, Konstantinos Zeimpekis, Hasan Sari, Lorenzo Nardo, Federico Caobelli, Ian Alberts, Axel Rominger, Thomas Pyka

**Affiliations:** 1 Department of Nuclear Medicine, Inselspital, University Hospital Bern, University of Bern, Freiburgstr. 18, 3010 Bern, Switzerland; 2 Advanced Clinical Imaging Technology, Siemens Healthcare AG, Lausanne, Switzerland; 3 Department of Radiology, University of California Davis, Sacramento, CA USA; 4 Molecular Imaging and Therapy, BC Cancer Agency, Vancouver, BC Canada

**Keywords:** Whole-body PET/CT, LAFOV PET/CT, Image quality, High blood glucose, Contrast-to-noise ratio

## Abstract

**Purpose:**

High blood glucose (hBG) in patients undergoing [^18^F]FDG PET/CT scans often results in rescheduling the examination, which may lead to clinical delay for the patient and decrease productivity for the department. The aim of this study was to evaluate whether long-axial field-of-view (LAFOV) PET/CT can minimize the effect of altered bio-distribution in hBG patients and is able to provide diagnostic image quality in hBG situations.

**Materials and methods:**

Oncologic patients with elevated blood glucose (≥ 8.0 mmol/l) and normal blood glucose (< 8.0 mmol/l, nBG) levels were matched for tumor entity, gender, age, and BMI. hBG patients were further subdivided into two groups (BG 8–11 mmol/l and BG > 11 mmol/l). Tracer uptake in the liver, muscle, and tumor was evaluated. Furthermore, image quality was compared between long acquisitions (ultra-high sensitivity mode, 360 s) on a LAFOV PET/CT and routine acquisitions equivalent to a short-axial field-of-view scanner (simulated (sSAFOV), obtained with high sensitivity mode, 120 s). Tumor-to-background ratio (TBR) and contrast-to-noise ratio (CNR) were used as the main image quality criteria.

**Results:**

Thirty-one hBG patients met the inclusion criteria and were matched with 31 nBG patients. Overall, liver uptake was significantly higher in hBG patients (SUV_mean_, 3.07 ± 0.41 vs. 2.37 ± 0.33; *p* = 0.03), and brain uptake was significantly lower (SUV_max_, 7.58 ± 0.74 vs. 13.38 ± 3.94; *p* < 0.001), whereas muscle (shoulder/gluteal) uptake showed no statistically significant difference. Tumor uptake was lower in hBG patients, resulting in a significantly lower TBR in the hBG cohort (3.48 ± 0.74 vs. 5.29 ± 1.48, *p* < 0.001). CNR was higher in nBG compared to hBG patients (12.17 ± 4.86 vs. 23.31 ± 12.22, *p* < 0.001). However, subgroup analysis of nBG 8–11 mmol/l on sSAFOV PET/CT compared to hBG (> 11 mmol/l) patients examined with LAFOV PET/CT showed no statistical significant difference in CNR (19.84 ± 8.40 vs. 17.79 ± 9.3, *p* = 0.08).

**Conclusion:**

While elevated blood glucose (> 11 mmol) negatively affected TBR and CNR in our cohort, the images from a LAFOV PET-scanner had comparable CNR to PET-images acquired from nBG patients using sSAFOV PET/CT. Therefore, we argue that oncologic patients with increased blood sugar levels might be imaged safely with LAFOV PET/CT when rescheduling is not feasible.

**Supplementary information:**

The online version contains supplementary material available at 10.1007/s00259-024-06646-5.

## Introduction

Positron emission tomography/computed tomography (PET/CT) has been established for tumor imaging and alters staging and treatment management in nearly 40% of the patients [1, 2]. Increased glycolysis and metabolism rate has been described in different tumor types allowing to use [^18^F]Fluorodeoxyglucose ([^18^F]FDG) as the main oncologic PET tracer [3]. [^18^F]FDG is transported by glucose transport proteins (GLUT) together with glucose in cells and after phosphorylation by the hexokinase trapped as [^18^F]FDG-6-phosphate [4]. Different GLUT subtypes with high glucose affinity (class I including GLUTs 1–4) have been identified. Their differing *K*_m_ (Michaelis constant, substrate concentration at half the maximum velocity) partly predicts their different functions [5]. GLUT 1 and 3, which are overexpressed in tumor and brain tissue, have low *K*_m_ (1–2 mmol/l) resulting in low *V*_max_, whereas GLUT 2 and 4 which are expressed in the liver and muscles have high *K*_m_ (GLUT 2, ~ 20 mmol/l; GLUT 4, 5 mmol/l) resulting in high *V*_max_ [6].

In conditions with elevated fasting blood glucose (BG) (> 8.0 mmol/l), it is assumed that GLUTs, especially with a low *K*_m_ (e.g., GLUT 1 in tumor cells and GLUT 3 in brain tissue), are saturated. Consecutively, hyperglycaemia can result in reduced [^18^F]FDG uptake in the tumor and brain as it competes with the endogenous glucose excess. Furthermore, sink effect can occur if the bio-distribution of [^18^F]FDG is altered [7].

Eskian et al. reported that the liver as the key organ for glucose regulation showed increased [^18^F]FDG uptake in hyperglycaemia [3]. Furthermore, brain uptake was significantly reduced in patients with elevated blood glucose. Moreover, GLUTs can be insulin-dependent (e.g., GLUT 4 in skeletal muscle), which may result in reduced [^18^F]FDG uptake in patients with diabetes due to an insulin resistance of the cells [3].

Many patients scheduled for an [^18^F]FDG PET/CT examination present with elevated BG levels due to an increasing incidence of pre-diabetic and diabetic disorders [8]. Therefore, the Societies of Nuclear Medicine in the USA (SNMMI) and in Europe (EANM) suggest to either delay the PET/CT examination or inject short acting insulin or hydrate the patient in cases of elevated BG (8.3–11.1 mmol/l and > 11.1 mmol/l) or reschedule the scan [9, 10]. An upper blood glucose limit of 8.9 mmol/l for [^18^F]FDG brain imaging was defined by Varrone et al. [11]. To date, there is uncertainty about the effect of the elevated BG on tumor uptake and background activity [12]. While some authors showed that tissue uptake (e.g., lung, bone marrow, spleen, fat, bowel and stomach) was not affected by high plasma glucose levels prior to [^18^F]FDG injection, whereas muscle uptake was slightly increased in hyperglycaemia [13]. Other works reported that tumor uptake is not significantly impaired by elevated blood glucose levels [3, 13–16].

In the last decade, digital detector technology and enhanced time-of-flight capabilities have resulted in significant improvements in PET sensitivity and resolution [17]. Further advancements were achieved by the recent introduction of long-axial field-of-view (LAFOV) PET-scanners with axial lengths over 100 cm [17–20]. With increasing length of the scanner, more lines of response (LOR) emitted from the patient hit the detectors [21], resulting in noise reduction and an improvement in sensitivity which may improve lesion conspicuity [19, 22–24]. These advantages might be clinically useful when image quality is expected to be impaired, as in hyperglycaemic patients receiving [^18^F]FDG.

In this study, we aim to evaluate the improvement of image quality when using a LAFOV PET system in patients with elevated blood glucose and aim to investigate whether this allows to compensate to some degree the altered distribution of [^18^F]FDG in elevated blood glucose situations.

## Materials and methods

### Patient population, radiopharmaceutical, and imaging

Nine hundred thirty-three oncologic patients undergoing clinical routine [^18^F]FDG-PET/CT on a LAFOV PET scanner were retrospectively evaluated. As per clinical routine, patients fasted for at least 6 h prior to the [^18^F]FDG-PET/CT. Blood glucose levels were confirmed by blood glucose sampling. In total, 3.0 ± 0.19 MBq/kg [^18^F]FDG was injected intravenously. All patients received skull base to mid thighs PET scans on the Biograph Vision Quadra (Siemens Healthineers) LAFOV PET/CT system. A 360 s list-mode (LM) acquisition was utilized for all scans.

### Patient characteristics and matched pair recruitment

**Fig. 1:**
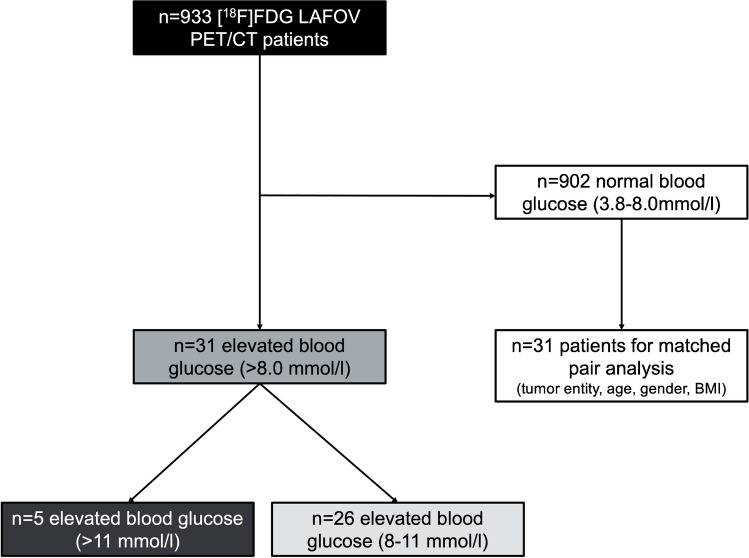
Study flowchart showing patient recruitment and distribution of blood glucose elevation

Thirty-one of 933 consecutively scanned patients showed elevated blood glucose levels (> 8.0 mmol/l) prior to the [^18^F]FDG injection. Patients were ask to refrain antidiabetic medication at least 72 h prior to the PET/CT. A total of 5/31 patients (16%) had blood glucose levels higher than 11 mmol/l, while 26/31 (84%) patients had blood glucose levels of 8–11 mmol/l. Patients with normal blood glucose levels (3.8–8.0 mmol/l) were matched to the group of high blood glucose patients with regard to baseline tumor entity, age, gender, and body mass index (BMI). Figure 1 shows the study flow-chart. Patients’ characteristics showing the different tumor features, which were included in the analysis, are outlined in Table 1.

**Table 1:** High blood glucose (hBG) and normal blood glucose (nBG) patients were matched with regard to the tumor entity (*n* = 31 per group). Patient cohorts include melanoma, lung cancer, lymphoma, breast cancer, genitourinary cancer (GU), head and neck cancer (HNC), and gastrointestinal cancer (GI). Patients’ characteristics (mean ± SD) as age (years), body mass index (BMI), normalized activities, gender, and blood glucose levels (mmol/l) are given

Cathegory	Subcathegory	High blood glucose (hBG), *n* = 31	Normal blood glucose (nBG), *n* = 31	***p*-**value
Tumor	Melanoma	10	10	1.00
	Lung cancer	9	9	
	Lymphoma	4	4	
	Breast cancer	1	1	
	GU cancer	2	2	
	HNC	3	3	
	GI cancer	2	2	
Age (years)	-	70 ± 9	66 ± 13	0.11
BMI (kg/m^2^)	-	28.22 ± 5.35	26.25 ± 4.53	0.20
Activity (MBq/kg)	-	3.01 ± 0.19	3.03 ± 0.07	0.70
Gender	Female	11	11	1.00
	Male	20	20	
Blood glucose (mmol/l)		**9.75 ± 1.39**	**6.08 ± 0.90**	** < 0.0001**

### Imaging protocol

The PET LM data were adapted using 360 s, 240 s, 120 s, and 60 s durations to simulate shorter acquisition times. The images were reconstructed using the ultra-high sensitivity mode (UHS, maximum ring difference MRD 322). To simulate the statistics and the image quality of a short-axial field-of-view PET-system (sSAFOV), the LM data were further reconstructed with 120 s and 60 s duration in the high sensitivity mode (HS, MRD 85) as previously described [24, 25]. The choice of sSAFOV was based on the coincident yield and sensitivity profile of the Biograph Vision Quadra to ensure reliable comparison between a SAFOV and LAFOV scanner [25, 26]. These reconstructions were used to compare tumor uptake, tumor-to-background ratio (TBR), and image quality between a LAFOV and a sSAFOV PET in hBG and nBG patients [22].

Image reconstruction was performed using the vendor’s standard reconstruction software (e7-tools, Siemens Healthineers), with OSEM (4 iterations, 5 subsets), point-spread-function (PSF), and TOF enabled. Images were reconstructed with a 440 × 440 × 644 image matrix with a voxel size of 1.65 × 1.65 × 1.65 mm^3^. A Gaussian post-reconstruction filter with 2-mm full width at half maximum (FWHM) was applied to the images. For attenuation correction, low-dose non-enhanced CT data were used. 3D scatter correction was performed using a 3D residual-based method which was especially developed for the MRD 322 [27] both for MRD 85 and 322 images [28].

CT scans were performed with equivalent parameters with slice thickness of 1.0 mm, pitch factor 1, bone and soft tissue reconstruction kernels, and maximum of 120 kV and 90 mAs by applying CARE kV and CARE Dose as previously published [22].

### Image evaluation

Three nuclear medicine physicians (CM, LW, and TP) performed image evaluation. For image evaluation, appropriate workstation [29] and software for quantitative image analysis and identification of target lesion were used (Syngo.via, Siemens Healthineers, Erlangen, Germany). Tumor-lesion uptake and metabolic tumor volumes were calculated by placing a volume-of-interest (VOI) with a 40%-iso-contour approach around the lesion as previously described [22, 30].

Peak and maximum standardized uptake values (SUV_peak/max_) were used to evaluate target lesion knowing that SUV_peak_ has been shown to be less susceptible for variation at different acquisition duration compared to SUV_max_ [31]. Lesions were evaluated at different scan times as LM data allowed different reconstructions (HS, 60 s, 120 s; UHS, 60 s, 120 s, 240 s, 360 s) [22, 32].

The background activity was measured by placing a 10-cm^3^ spherical VOI in the right lobe of healthy liver tissue as previously described [33]. The VOIs were copied to same anatomical locations in all different images obtained from different (list-mode) frame durations within the same patient to ensure that mistakes can be minimized. The coefficient of variation (COV) for the liver background was defined by the following formula where *σ* was the standard deviation of the liver VOI and *μ* was the SUV_mean_ of the liver VOI [22]. TBR was defined as tumor uptake (SUV_max_) per liver uptake (SUV_mean_). Contrast-to-noise ratio (CNR) was calculated as previously published [34].

$$COV\;=\;(\frac{\sigma\;}{\mu\;});\;TBR=\frac{SUVmax\;(tumor)}{SUV\;mean\;(liver)};\;CNR\;=\;\frac{SUVmean\;\left(lesion\right)\;-\;SUVmean\;(background)}{SD\;(background)}$$

### Statistical analysis

Statistical analyses were performed using Excel (Microsoft, Redmond, Washington) and GraphPad Prism Version 8 (San Diego, CA) [35]. The data are presented either as mean ± standard deviation or as median and range. Qualitative comparison between hBG and nBG patients was analyzed using Fisher’s exact test, and for quantitative measurements (COV, CNR, SUV), were analyzed using unpaired Student’s *T*-test after verification of normal distribution. Comparison between different reconstructions in the same patient were analyzed using paired Student’s *T*-test and Bonferroni-correction was applied. *p*-values < 0.05, in cased of Bonferroni-corrections *p* < 0.001, were considered statistically significant.

## Results

Overall, 78 nBG and 82 hBG tumor lesions were identified. The metabolic tumor volume (MTV) and total lesion glycolysis (TLG) did not differ significantly between both patient groups (median, range; hBG MTV, 0.94 ml, 0.01–7.45 ml vs. nBG, 1.23 ml, 0.12–18.48 ml; *p* = 0.06 and hBG TLG, 7.02 ml, 0.6–749.75 ml vs. nBG, 4.43 ml, 0.53–181.61 ml; *p* = 0.24). In the hBG patients, 70 lesions (85%) were detected in the subgroup of patients with BG 8–11 mmol/l and 12 (15%) in the subgroup of patients with BG > 11 mmol/l.

### Background activity (liver and muscle uptake)

**Fig. 2:**
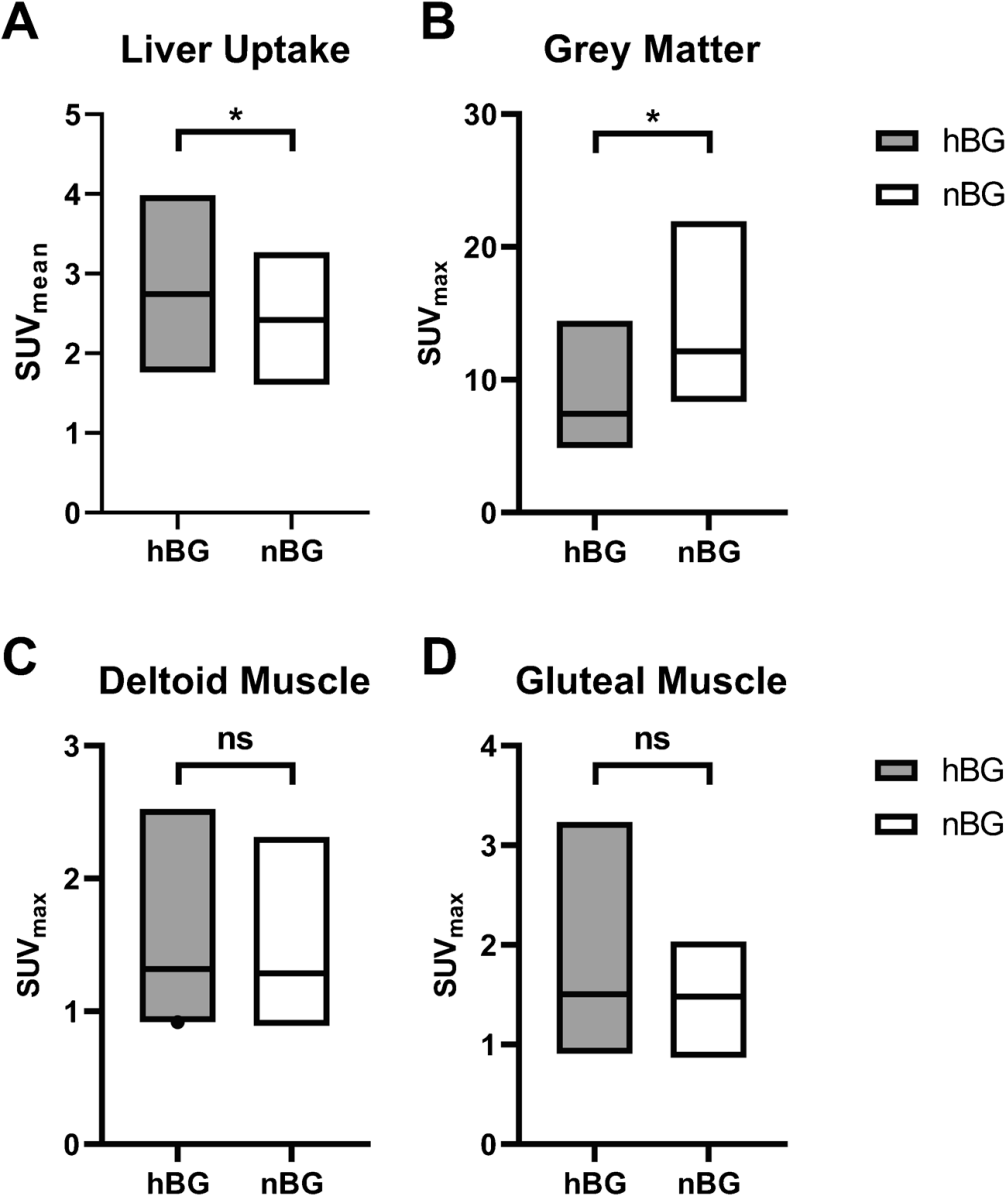
Background uptake visualized with box plots in patients with high (hBG) and normal blood glucose (nBG). Shown are standardized uptake value (SUV_mean_) of liver uptake (**A**), SUV_max_ of brain (gray matter, **B**), and muscle uptake (shoulder, **C**; gluteal, **D**). Statistically significant differences are highlighted with an asterisk “*”; ns, not statistically significant

Liver uptake in hBG patients was significantly higher compared to nBG patients (SUV_mean_, 3.07 ± 0.41 vs. 2.37 ± 0.33; *p* = 0.03) whereas the brain (gray matter) uptake was significantly lower in hBG patients compared to nBG patients (SUV_max_, 7.58 ± 0.74 vs. 13.38 ± 3.94; *p* < 0.001 and SUV_peak_, 5.70 ± 0.25 vs. 9.91 ± 3.46; *p* < 0.001) (Fig. 2A, B).

Muscle uptake was not statistically different between the hBG and nBG cohort neither in shoulder nor in gluteal muscle (shoulder SUV_max_, 1.63 ± 0.47 vs. 2.09 ± 0.26; *p* = 0.91 and gluteal SUV_max_, 1.47 ± 0.18 vs. 1.45 ± 0.12; *p* = 0.33) (Fig. 2C, D).

### Tumor uptake in hBG and nBG patients

**Fig. 3:**
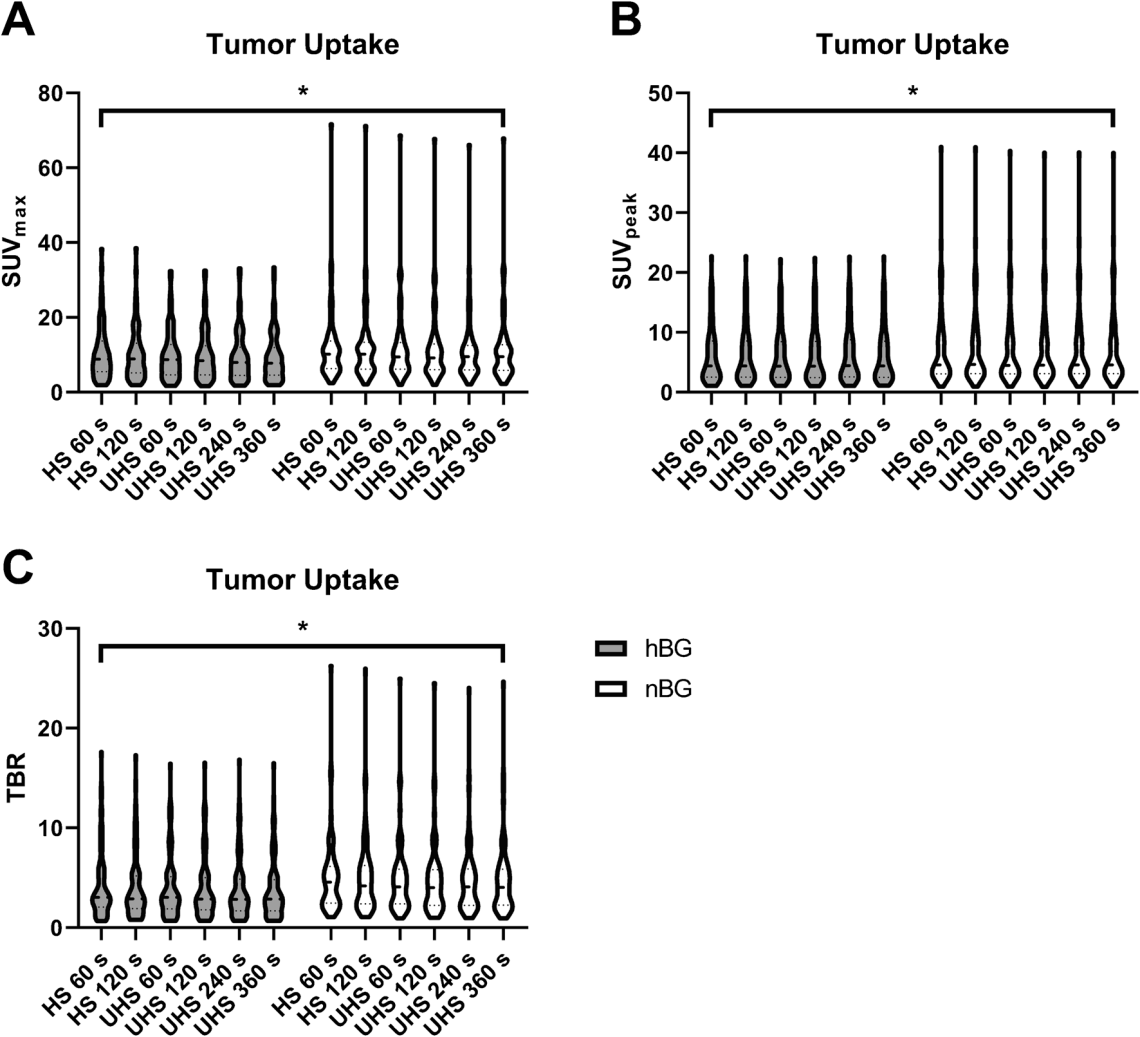
Tumor uptake visualized with violin plots in patients with high (hBG) and normal blood glucose (nBG) (**A**–**C**). Shown are standardized uptake values (SUV_max/peak_) and tumor to background ratios (TBR). Statistically significant differences are highlighted with an asterisk “*”

In all reconstructions (HS 60 s, HS 120 s, UHS 60 s, UHS 120 s, UHS 240 s, and UHS 360 s), hBG patients had slightly, but still significantly lower tumor uptake compared to the matched-pair equivalent with nBG (SUV_max_ hBG, 11.05 ± 2.29 vs. nBG, 12.83 ± 5.35; *p* < 0.001; SUV_peak_ hBG, 6.36 ± 2.16 vs. nBG, 8.85 ± 5.03; *p* < 0.001). TBR between hBG and nBG reconstructions was consecutively significantly lower in hBG patients (TBR hBG, 3.48 ± 0.74 vs. nBG, 5.29 ± 1.48; *p* < 0.001) (Fig. 3).

The results were confirmed in the subgroup analysis. Tumor uptake was lower in both subgroups compared to the nBG patients. Consecutively, TBR was significantly lower in both subgroups compared to nBG patients (TBR BG, 8–11 mmol/l, 3.88 ± 3.24 vs. nBG, 4.73 ± 3.87; *p* < 0.0001 and BG > 11 mmol/l, 4.11 ± 0.89 vs. nBG, 5.25 ± 1.42; *p* < 0.0001). Detailed visualization of the subgroup results can be found in the Supplementary Material.

### Background noise (COV) and image quality (CNR)

**Fig. 4:**
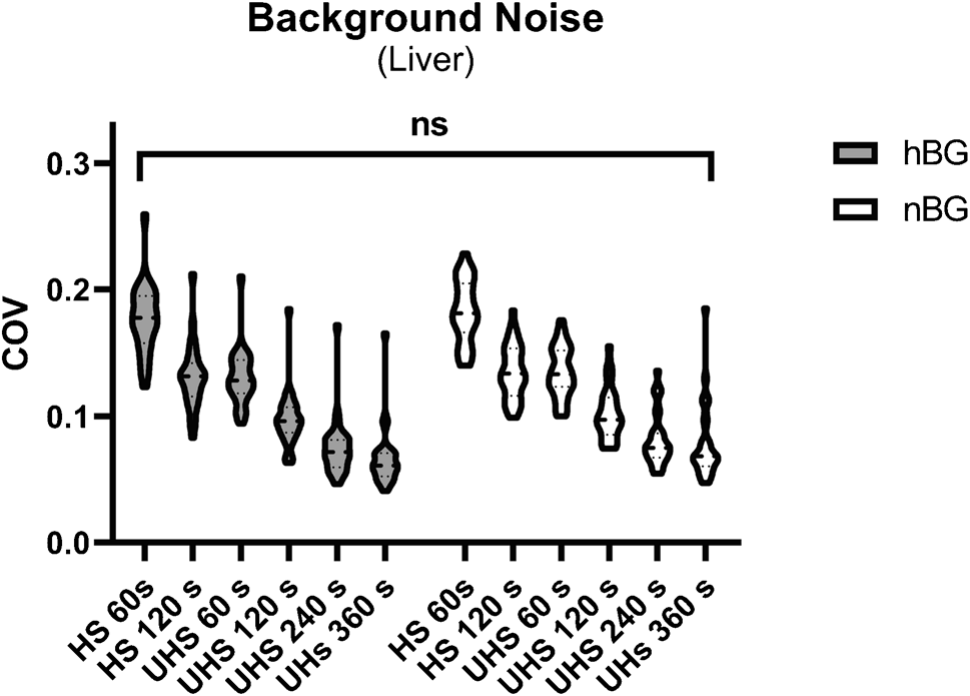
Background noise of the liver visualized with violin plots in patients with high (hBG) and normal blood glucose (nBG). Shown is the coefficient of variation (COV) in liver tissue. Brackets summarize the comparison between each reconstruction in hBG and nBG and indicate that differences were not statistically significant (ns) between all corresponding reconstructions

In order to compare background noise, liver COV in the hBG and nBG group was determined. We found no statistical difference between the COV of each reconstruction comparing hBG vs. nBG patients (COV hBG, 0.09 ± 0.39 vs. nBG, 0.10 ± 1.23; *p* = 0.10) (Fig. 4). COV decreased with acquisition time as with lowest COV after 360 s acquisition in UHS mode (hBG, 0.07 ± 0.37 vs. nBG, 0.07 ± 0.86; *p* = 0.16). All obtained reconstructions showed comparable COV in situations of high and normal blood glucose.

**Fig. 5:**
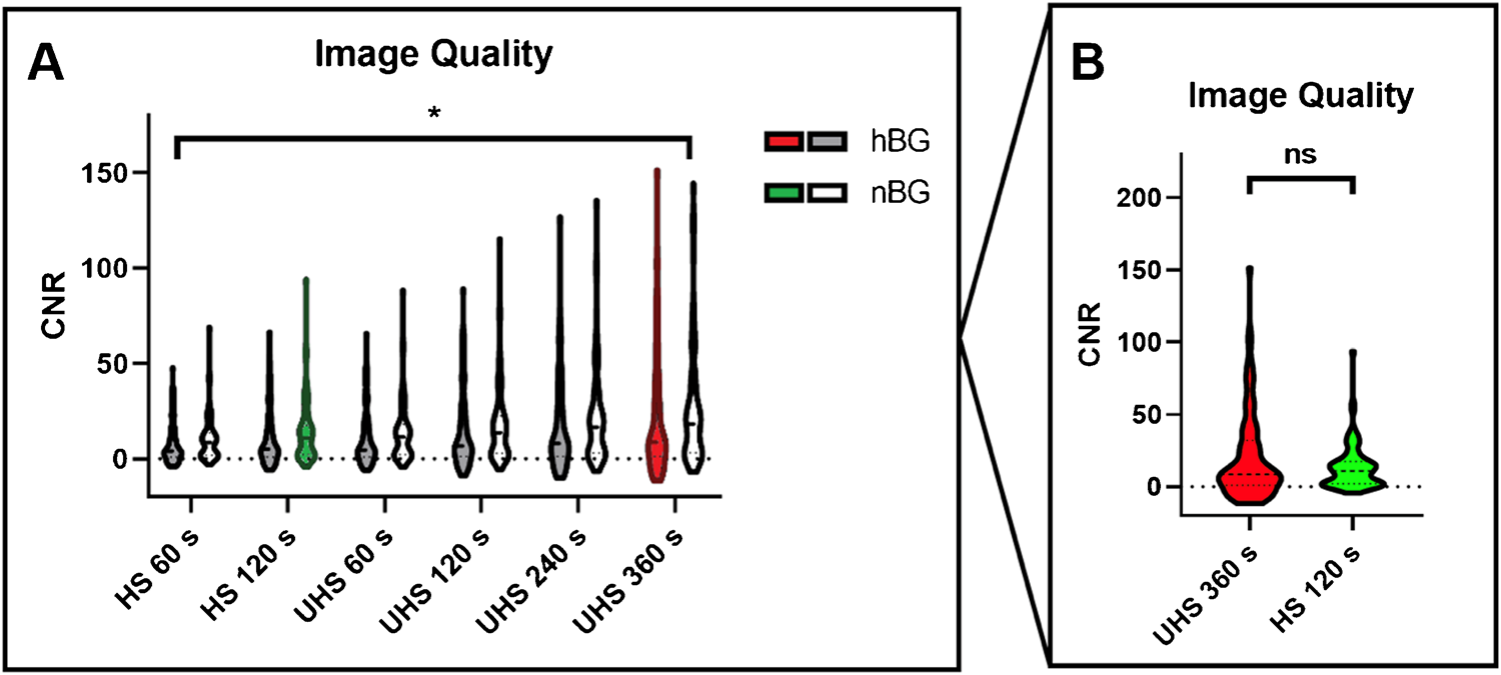
Image quality of all reconstructions between high (hBG) and normal blood glucose patients (nBG). Visualized are contrast to noise ratios (CNR) in all reconstructions (**A**). **B** Chosen reconstructions for comparison UHS 360 s for LAFOV PET and HS 120 s for sSAFOV PET. Statistically significant differences are highlighted with an asterisk “*”; ns, not statistically significant. Brackets summarize the comparison between each reconstruction in hBG and nBG and indicate that differences were significant between all corresponding reconstructions

With regard to CNR, we noted that CNR behaved similar to the tumor uptake and was significantly higher in nBG compared to hBG comparing corresponding reconstructions (CNR hBG, 12.17 ± 4.86 vs. nBG, 23.31 ± 12.22; *p* < 0.001) (Fig. 5A). However, CNR in nBG patients receiving sSAFOV (HS 120 s, Fig. 5A (green)) and hBG patients receiving LAFOV (UHS 360 s, Fig. 5B (red)) was similar (sSAFOV nBG, 17.79 ± 9.3 vs. LAFOV hBG, 19.84 ± 8.4; *p* = 0.08).

### Subgroup analysis with regard to tumor uptake and image quality

**Fig. 6:**
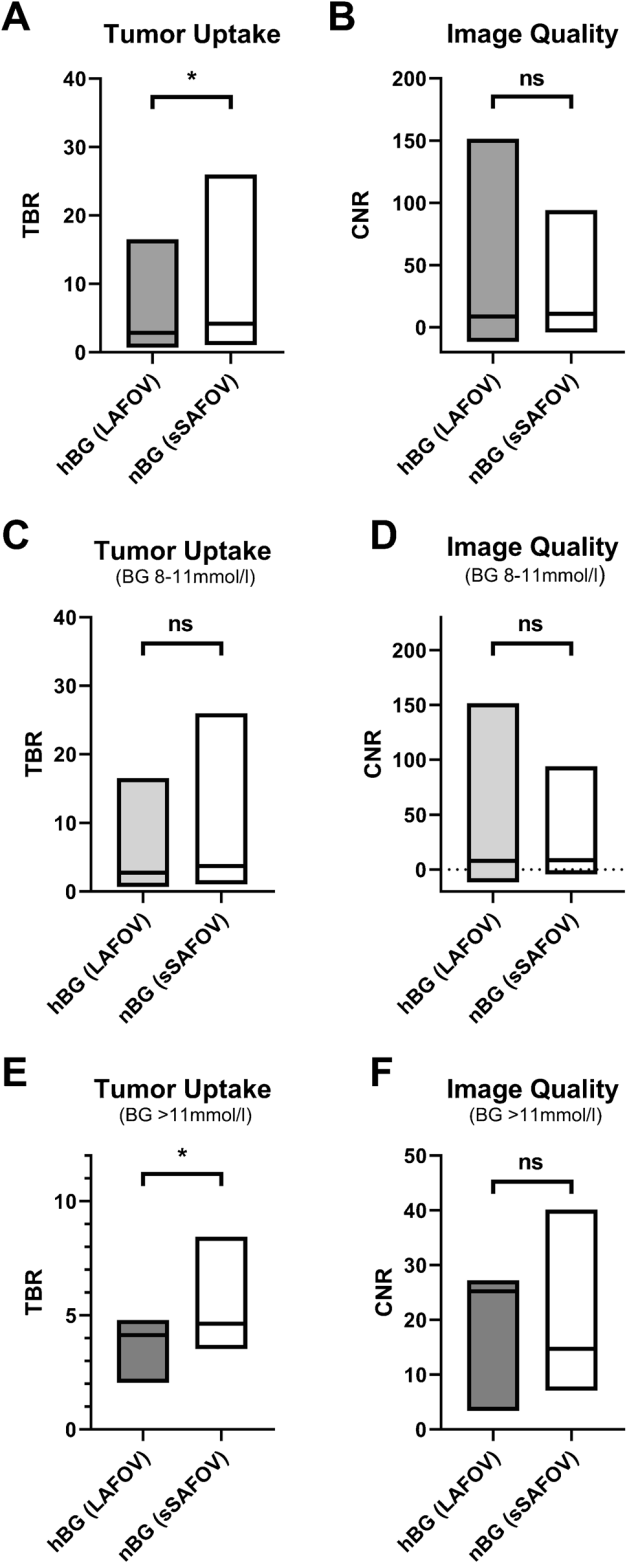
Tumor uptake und image quality in comparison of long-axial field-of-view (LAFOV) high blood glucose (hBG, **A**) and simulated short-axial field-of-view (sSAFOV) normal blood glucose (nBG, **B**) patients. Box plots (**C**, **D**) show the subgroup with BG 8–11 mmol/l and (**E**, **F**) BG > 11 mmol/l. Statistically significant differences are highlighted with an asterisk “*”; ns, not statistically significant

Subgroup analysis of patients with hBG 8–11 mmol/l revealed no statistical difference in comparison of hBG LAFOV (UHS 360 s) vs. nBG sSAFOV (HS 120 s) reconstructions neither in TBR nor in CNR (TBR hBG LAFOV, 3.70 ± 3.12 vs. nBG sSAFOV, 4.86 ± 3.95; *p* = 0.06 and CNR hBG LAFOV, 20.59 ± 31.92 vs. nBG sSAFOV, 13.10 ± 16.34; *p* = 0.09). This finding was consistent with the overall analysis, which did not reveal statistical differences between the CNR of hBG LAFOV and nBG sSAFOV scans (*p* = 0.08) (Fig. 6).

**Fig. 7:**
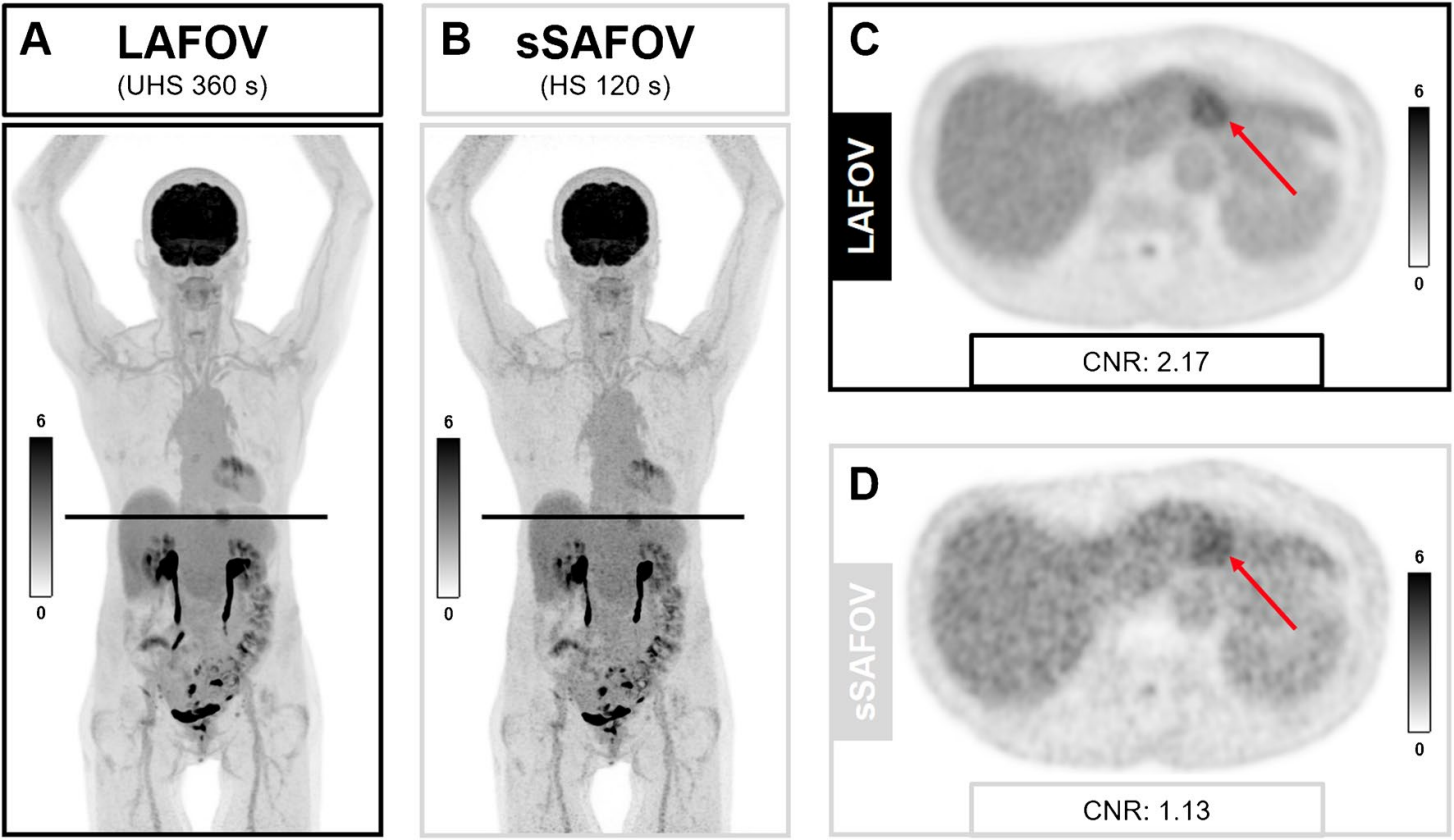
Example SUV image of a 46 year/o female patient referred to our PET/CT center for restaging of endometrial cancer. Prior to the [^18^F]FDG scan, elevated blood glucose was elevated (BG, 9.4 mmol/l). In this study, a new liver metastasis was detected (marked with a red arrow). Shown are maximum intensity projections (**A**, **B**) and axial images (**C**, **D**) of long-axial field-of-view (LAFOV, **A**, **C**) and simulated short-axial field-of view (sSAFOV (**B**, **D**)) PET-reconstructions. Of note, contrast-to-noise ratio (CNR) of the liver lesion was significantly higher in the LAFOV compared to sSAFOV images. Scale bars indicate the SUV window used for this patient

In patients with hBG > 11 mmol/l, we found different results. The TBR was significantly lower in hBG LAFOV compared to nBG sSAFOV (TBR hBG LAFOV, 3.8 ± 0.9 vs. nBG sSAFOV, 5.32 ± 1.41; *p* = 0.03), whereas the CNR was not different in the hBG 8–11 mmol/l group between hBG LAFOV compared to nBG sSAFOV (CNR hBG LAFOV, 20.6 ± 8.4 vs. nBG sSAFOV, 17.10 ± 9.15; *p* = 0.46) (Fig. 6). An example image of hBG patients shows that CNR is significantly higher in LAFOV compared to sSAFOV (Fig. 7).

## Discussion

The rising prevalence of pre-diabetic and diabetic patients results in an increasing portion of patients with elevated blood glucose levels despite fasting 6 h prior to the [^18^F]FDG injection [8]. As described above, hBG leads to altered [^18^F]FDG uptake in both healthy tissue and neoplasms, resulting in a generally reduced image quality. The recently introduced LAFOV PET/CT scanners enable performing scans with higher sensitivity and lower noise as well as higher lesion conspicuity [22, 24, 36]. We present the first matched pair analysis including patients with hBG and nBG comparing the background, liver, tumor uptake, and image quality on LAFOV and sSAFOV PET/CT.

Our results are in keeping with previous reports on [^18^F]FDG bio-distribution. We found that liver uptake (SUV_mean_) was significantly higher in hBG patients compared to nBG patients. That was consistent with the findings of Lindholm et al. who report a significant correlation between increased BG levels and liver uptake using SUV_mean_ (correlation coefficient, 0.12; *p* < 0.001) [13]. Furthermore, Büsing et al. report a negative correlation between patients with hBG and nBG in brain uptake (Pearson coefficient, − 0.44; *p* < 0.01); this is congruous with our findings as we found significantly decreased brain uptake in the cohort of hBG patients. This effect of elevated BG in [^18^F]FDG PET/CT resulted in the recommendation of an upper BG limit of 8.9 mmol/l for [^18^F]FDG brain imaging (e.g., dementia, epilepsy) [14, 15].

Our cohort showed no significant difference in muscle uptake (neither deltoid nor gluteal) in hBG and nBG patients. Thus, we found a tendency toward increased uptake in the gluteal muscle in hBG patients, but not statistically significant (SUV_max_, 1.47 ± 0.18 vs. 1.45 ± 0.12; *p* = 0.33). Previous publications report discrepant results. Lindholm et al. and Büsing et al. showed a weak positive correlation between BG levels and muscular [^18^F]FDG uptake, whereas Eskin et al. report different findings. Muscular SUV_max_ was inversely correlated with increasing BG and SUV_mean_ showed no significant correlation to serum BG [3]. Accordingly, Webb et al. found no significant difference in muscle uptake between patients with nBG and hBG [37]. As possible confounders, gender, age, BMI, diabetes, [^18^F]FDG dose, and scan time were identified. We tried to avoid those inconsistencies using a matched pair study design and matching with regard to tumor entity, gender, age, and BMI. Still, we noticed that patients with hBG had higher BMI in our cohort compared with nBG patients (28.22 ± 5.35 kg/m^2^ vs. 26.25 ± 4.53 kg/m^2^) [38].

Our cohort of hBG patients showed significantly lower tumor uptake compared to the nBG cohort over all reconstructions and consecutively significantly lower TBR. Subgroup findings were consistent with overall findings. These findings are perfectly in line with the literature. The majority of publications show that tumor [^18^F]FDG uptake decreases with increasing serum BG [3, 14, 37]. Moreover, significant TBR reduction is the consequence of decreased tumor and increased liver uptake. That is the reason why SNMMI and EANM guidelines suggest to reschedule [^18^F]FDG PET/CT in cases of elevated serum BG [9, 10].

Rescheduling the PET/CT examination leads to increased cost for the healthcare system, inconvenience for the patients, and can result in an unnecessary delay of the tumor staging which may affect the therapy of the patient [3]. Therefore, it is important, especially for oncologic patients to avoid unnecessary delay of the PET/CT. We tried to address this problem by analyzing the image quality of [^18^F]FDG on a LAFOV compared to a (simulated) SAFOV scanner in cases of high blood glucose. sSAFOV scan parameters were determined by the performance characteristics of the Biograph Vision Quadra PET-system. Prenosil et al. reported by evaluating the performance characteristics of the Biograph Vision Quadra according to the National Electrical Manufacturers Association NU 2–2018 (NEMA) standard increased sensitivity and noise equivalent count rates (NECR), which were by 5 and 10 times higher compared to a SAFOV system (Biograph Vision 600) in MRD 85 and MRD 322 mode, respectively [25]. Further publication with patient data showed that a scan with an acquisition time of 120 s in MRD 85 was equivalent comparing to a the mentioned SAFOV system [22]. Moreover, further increase in sensitivity and NECRs was reported with the use of the LAFOV PET/CT’s full acceptance angle (MRD 322) resulting in the possibility to reduce acquisition times and injected doses even further compared to the SAFOV system [24].

In the present work, we showed that the tumor uptake and TBR decrease with increasing BG on a LAFOV scanner, which is consistent to the published data. Accordingly, overall CNR was significantly lower in hBG compared to nBG patients. However, background noise was not significantly different between hBG and nBG patients. Moreover, and most importantly, hBG patients receiving LAFOV PET/CT showed comparable image quality (*p* = 0.08) as measured by CNR as that of matched nBG patients receiving sSAFOV reconstructions (Fig. 6).

Subgroup analysis revealed that tumor uptake differed only significantly in LAFOV compared to sSAFOV in patients with BG levels > 11 mmol/l. In patients with BG 8.0–11.0 mmol/l, no statistical significant difference was found between LAFOV and sSAFOV PET. Our data regarding the tumor uptake are in agreement with so far published findings and guideline recommendation. Eskian et al. found that only the group with BG > 11.1 mmol/l (Eq. 200 mg/dl) had significantly lower SUV compared to euglycemic patients and EANM guideline states that [^18^F]FDG PET/CT can be performed for clinical studies if plasma glucose is lower than 11 mmol/l [3, 9]. Furthermore, Boellaard et al. acknowledge that elevated BG can occur in situations of poorly controlled diabetes or can be associated with infection. Therefore, they argue that hyperglycaemia should not represent an absolute contraindication [9].

In our cohort, LAFOV PET/CT was able to obtain PET-image quality in situations with elevated (BG, 8–11 mmol/l) or highly elevated (> 11 mmol/l) BG compared to nBG patients scanned with SAFOV PET/CT (Fig. 6). An example of a subtle liver metastasis in a female patient with hBG shows how the image quality (CNR) improves significantly in LAFOV PET/CT compared to sSAFOV PET/CT (Fig. 7). We therefore argue that higher sensitivity and lower background noise in LAFOV PET/CT can compensate to some degree the altered bio-distribution of [^18^F]FDG in oncologic patients with elevated blood glucose. Nevertheless, SUV measurements in cases of elevated BG should be used carefully when comparison with follow-up PET-imaging is required. Patient reports should particularly mention this limitation to draw clinicians’ attention to the hBG PET-examination.

Furthermore, we note several limitations of our study. First, the matched-pair study design introduced some selection bias. We tried to address all necessary oncological features of each patient to adjust the cohorts as best as possible. Moreover, we matched each patient according to age, gender, and BMI. A prospective, preferably head-to-head study design would have avoided this bias. However, due to the retrospective nature of the study, a head-to-head evaluation was not possible, which would have required two independent scans on two different days leading to further delay of the oncological treatment. Secondly, we could not compare LAFOV with SAFOV PET/CT directly. Instead, we used reconstructions simulating SAFOV PET/CT [22, 24]. However, using sSAFOV enabled to perform an intra-individual comparison of all reconstructions and avoided further bias. Lastly, the matched-pair cohort was limited to a relatively small number of patients, which resulted from only 3.32% of investigated patients meeting the inclusion criteria. Many of our PET/CT examinations were delayed due to high BG levels. Nevertheless, possible non-[^18^F]FDG-avid lesions might have been missed, especially in situations of hBG patients.

With the limitations in mind, our data suggest that the higher sensitivity of LAFOV may compensate for the reduced CNR in cases of elevated blood glucose, and that in borderline clinical circumstances, rescheduling the study or maneuvres to reduced blood glucose levels may not be necessary.

## Conclusion

In a matched-pair analysis of patients with elevated blood glucose levels (> 8 mmol/l) and patients with normal blood glucose prior to examination with [^18^F]FDG PET/CT on a long axial field-of-view scanner, we found decreasing brain uptake, increasing background activity (e.g., liver uptake), and decreasing tumor uptake. However, we showed that LAFOV PET/CT could compensate to some degree the altered bio-distribution in hBG patients using a higher sensitivity profile and a lower noise rate compared to SAFOV PET/CT.

We, therefore, argue that using a LAFOV PET/CT might be advantageous in patients with high BG levels when decisions to delay imaging would lead to increased costs, inconvenience for the patient, and delay in oncological treatment. In such cases, especially if BG is ≥ 11 mmol/l, we recommend performing [^18^F]FDG LAFOV PET/CT imaging with longer acquisition duration whenever possible.

### Supplementary information

Below is the link to the electronic supplementary material.

Supplementary file1 (DOCX 806 KB)

## Data Availability

The data are available upon request at the corresponding author’s address.

## References

[1] Beyer T, Townsend DW, Brun T, Kinahan PE, Charron M, Roddy R (2000). A combined PET/CT scanner for clinical oncology. Journal of nuclear medicine: official publication, Society of Nuclear Medicine.

[2] Gambhir SS, Czernin J, Schwimmer J, Silverman DH, Coleman RE, Phelps ME (2001). A tabulated summary of the FDG PET literature. Journal of nuclear medicine: official publication, Society of Nuclear Medicine.

[3] Eskian M, Alavi A, Khorasanizadeh M, Viglianti BL, Jacobsson H, Barwick TD (2019). Effect of blood glucose level on standardized uptake value (SUV) in 18F- FDG PET-scan: a systematic review and meta-analysis of 20,807 individual SUV measurements. Eur J Nucl Med Mol Imaging.

[4] Pauwels EK, McCready VR, Stoot JH, van Deurzen DF (1998). The mechanism of accumulation of tumour-localising radiopharmaceuticals. Eur J Nucl Med.

[5] Michaelis L, Menten ML, Johnson KA, Goody RS (2011). The original Michaelis constant: translation of the 1913 Michaelis-Menten paper. Biochemistry.

[6] Gorovits N, Charron MJ (2003). What we know about facilitative glucose transporters: lessons from cultured cells, animal models, and human studies. Biochem Mol Biol Educ.

[7] Clark MS, Packard AT, Johnson DR, Johnson GB (2019). Pitfalls of a mixed metabolic response at PET/CT. Radiographics.

[8] Khan MAB, Hashim MJ, King JK, Govender RD, Mustafa H, Al KJ (2020). Epidemiology of type 2 diabetes - global burden of disease and forecasted trends. J Epidemiol Glob Health.

[9] Boellaard R, Delgado-Bolton R, Oyen WJ, Giammarile F, Tatsch K, Eschner W (2015). FDG PET/CT: EANM procedure guidelines for tumour imaging: version 2.0. European journal of nuclear medicine and molecular imaging..

[10] Delbeke D, Coleman RE, Guiberteau MJ, Brown ML, Royal HD, Siegel BA (2006). Procedure guideline for tumor imaging with 18F-FDG PET/CT 1.0. Journal of nuclear medicine: official publication, Society of Nuclear Medicine..

[11] Varrone A, Asenbaum S, Vander Borght T, Booij J, Nobili F, Någren K (2009). EANM procedure guidelines for PET brain imaging using [18F]FDG, version 2. Eur J Nucl Med Mol Imaging.

[12] Kawasaki K, Ishii K, Saito Y, Oda K, Kimura Y, Ishiwata K (2008). Influence of mild hyperglycemia on cerebral FDG distribution patterns calculated by statistical parametric mapping. Ann Nucl Med.

[13] Lindholm H, Brolin F, Jonsson C, Jacobsson H (2013). The relation between the blood glucose level and the FDG uptake of tissues at normal PET examinations. EJNMMI Res.

[14] Büsing KA, Schönberg SO, Brade J, Wasser K (2013). Impact of blood glucose, diabetes, insulin, and obesity on standardized uptake values in tumors and healthy organs on 18F-FDG PET/CT. Nucl Med Biol.

[15] Evangelista L, Gori S, Rubini G, Gallo M (2019). Management of hyperglycemia in oncological patients scheduled for an FDG-PET/CT examination. Clinical and Translational Imaging.

[16] Sprinz C, Altmayer S, Zanon M, Watte G, Irion K, Marchiori E (2018). Effects of blood glucose level on 18F-FDG uptake for PET/CT in normal organs: a systematic review. PLoS ONE.

[17] Vandenberghe S, Moskal P, Karp JS (2020). State of the art in total body PET. EJNMMI Physics.

[18] Pantel AR, Mankoff DA, Karp JS. Total body PET – will it change science and practice? Journal of Nuclear Medicine. 2022;jnumed.121.263481 10.2967/jnumed.121.263481. 10.2967/jnumed.121.263481 PMC12079751 35273091

[19] Badawi RD, Shi H, Hu P, Chen S, Xu T, Price PM (2019). First human imaging studies with the EXPLORER total-body PET scanner. Journal of nuclear medicine : official publication, Society of Nuclear Medicine.

[20] Mingels C, Caobelli F, Alavi A, Sachpekidis C, Wang M, Nalbant H (2023). Total-body PET/CT or LAFOV PET/CT? Axial field-of-view clinical classification. Eur J Nucl Med Mol Imaging.

[21] Eriksson L, Townsend D, Conti M, Eriksson M, Rothfuss H, Schmand M (2007). An investigation of sensitivity limits in PET scanners. Nucl Instrum Methods Phys Res, Sect A.

[22] Alberts I, Hünermund J-N, Prenosil G, Mingels C, Bohn KP, Viscione M (2021). Clinical performance of long axial field of view PET/CT: a head-to-head intra-individual comparison of the Biograph Vision Quadra with the Biograph Vision PET/CT. Eur J Nucl Med Mol Imaging.

[23] Karp JS, Viswanath V, Geagan MJ, Muehllehner G, Pantel AR, Parma MJ (2020). PennPET explorer: design and preliminary performance of a whole-body imager. Journal of nuclear medicine : official publication, Society of Nuclear Medicine.

[24] Mingels C, Weidner S, Sari H, Buesser D, Zeimpekis K, Shi K (2023). Impact of the new ultra-high sensitivity mode in a long axial field-of-view PET/CT. Ann Nucl Med.

[25] Prenosil GA, Sari H, Fürstner M, Afshar-Oromieh A, Shi K, Rominger A (2022). Performance characteristics of the biograph vision quadra PET/CT system with a long axial field of view using the NEMA NU 2–2018 standard. Journal of nuclear medicine : official publication, Society of Nuclear Medicine.

[26] Prenosil GA, Hentschel M, Weitzel T, Sari H, Shi K, Afshar-Oromieh A (2022). EARL compliance measurements on the biograph vision Quadra PET/CT system with a long axial field of view. EJNMMI Phys.

[27] Bal H, Panin VY, Cabello J, Schaefferkoetter J, Conti M. Fully 3D scatter estimation in axially long FOV PETCT scanners: residual estimation approach. 2021.

[28] Bal H, Aykac M, Conti M. <strong>A novel approach for scatter correction in PET using energy response modelling</strong>. Journal of Nuclear Medicine. 2020;61:108-.

[29] Mingels C, Sachpekidis C, Bohn KP, Hünermund JN, Schepers R, Fech V (2021). The influence of colour scale in lesion detection and patient-based sensitivity in [68Ga]Ga-PSMA-PET/CT. Nucl Med Commun.

[30] Lee H, Paeng JC, Hong SH, Yoo HJ, Cheon GJ, Lee DS (2016). Appropriate margin thresholds for isocontour metabolic volumetry of fluorine-18 fluorodeoxyglucose PET in sarcoma: a hybrid PET/MRI study. Nucl Med Commun.

[31] Sher A, Lacoeuille F, Fosse P, Vervueren L, Cahouet-Vannier A, Dabli D (2016). For avid glucose tumors, the SUV peak is the most reliable parameter for [(18)F]FDG-PET/CT quantification, regardless of acquisition time. EJNMMI Res.

[32] Sari H, Mingels C, Alberts I, Hu J, Buesser D, Shah V (2022). First results on kinetic modelling and parametric imaging of dynamic (18)F-FDG datasets from a long axial FOV PET scanner in oncological patients. Eur J Nucl Med Mol Imaging.

[33] Wahl RL, Jacene H, Kasamon Y, Lodge MA (2009). From RECIST to PERCIST: evolving considerations for PET response criteria in solid tumors. Journal of nuclear medicine : official publication, Society of Nuclear Medicine.

[34] Yan J, Schaefferkoetter J, Conti M, Townsend D (2016). A method to assess image quality for Low-dose PET: analysis of SNR, CNR, bias and image noise. Cancer Imaging.

[35] Mingels C, Bohn KP, Rominger A, Afshar-Oromieh A, Alberts I (2022). Diagnostic accuracy of [(18)F]PSMA-1007 PET/CT in biochemical recurrence of prostate cancer. Eur J Nucl Med Mol Imaging.

[36] Cherry SR, Jones T, Karp JS, Qi J, Moses WW, Badawi RD (2018). Total-body PET: maximizing sensitivity to create new opportunities for clinical research and patient care. Journal of nuclear medicine : official publication, Society of Nuclear Medicine.

[37] Webb RL, Landau E, Klein D, DiPoce J, Volkin D, Belman J, et al. Effects of varying serum glucose levels on 18F-FDG biodistribution. Nuclear medicine communications. 2015;36. 10.1097/MNM.0000000000000319 25888357

[38] Caobelli F, Pizzocaro C, Paghera B, Guerra UP (2013). Proposal for an optimized protocol for intravenous administration of insulin in diabetic patients undergoing (18)F-FDG PET/CT. Nucl Med Commun.

