# Supplementary material for: FDG imaging with long-axial field-of-view PET/CT in patients with high blood glucose—a matched pair analysis
Source: Eur J Nucl Med Mol Imaging. 2024 Feb 22;51(7):2036–46. doi: 10.1007/s00259-024-06646-5 (PMC11139721; doi:10.1007/s00259-024-06646-5)
Supplement: Supplementary file 1 — Supplementary file1 (DOCX 806 KB) [file 259_2024_6646_MOESM1_ESM.docx]

**Supplementary Material**


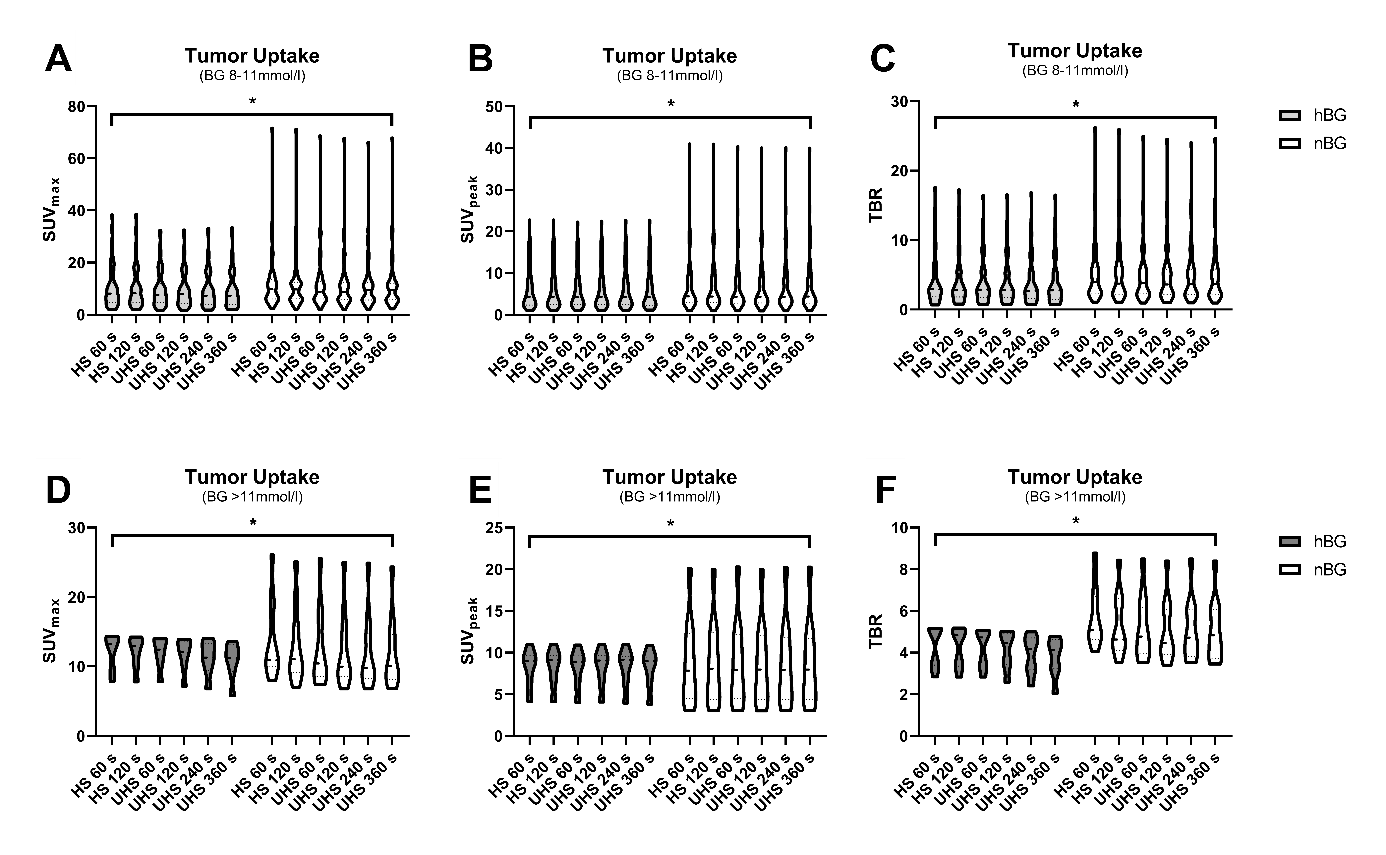


**Figure 1:** Tumour uptake in the hBG subgroups with blood glucose levels 8-11 mmol/l (A-C) and >11 mmol/l (D-F) visualized with violin plots compared to nBG patients. Shown are standardized uptake values (SUV_max/peak_) and tumour to background ratios (TBR). Statistically significant differences are highlighted with an asterisk “*”.


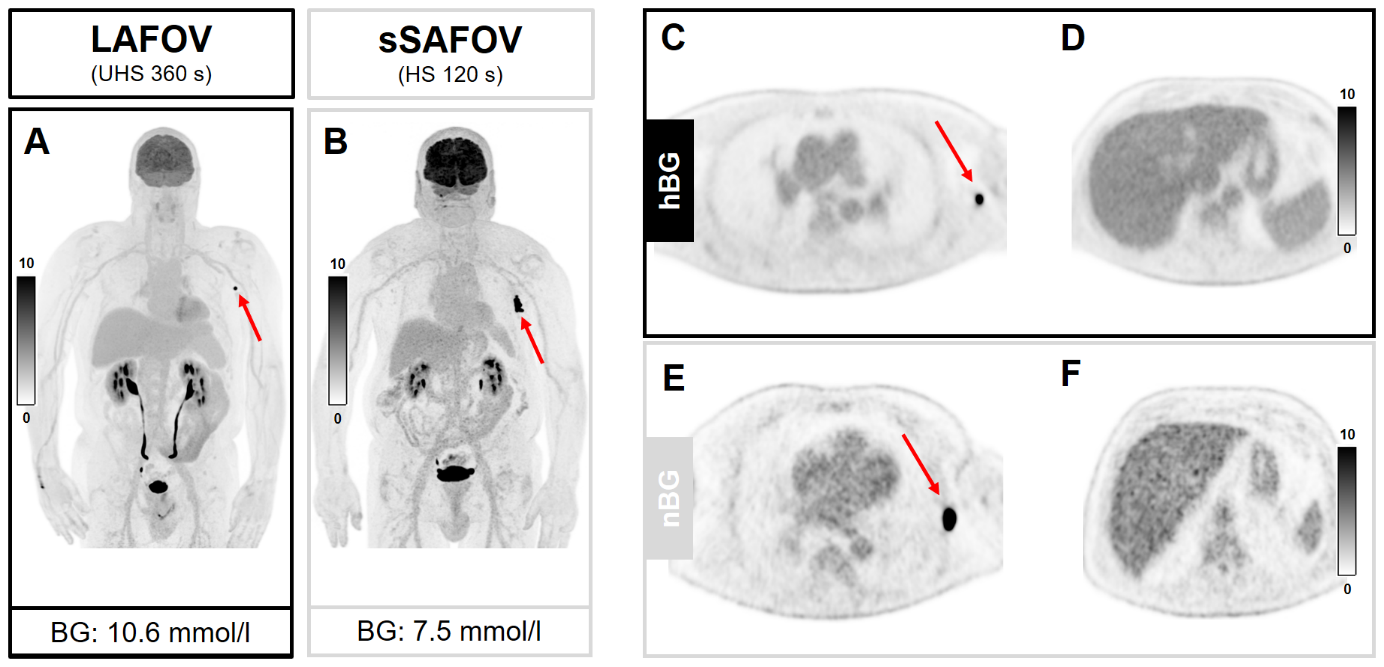


**Figure 2:** Shown are example maximum intensity projections (MIP, A/B) and axial standardized uptake value (SUV) PET images (C-F) of one hBG (A/C/D) and nBG (B/E/F) matched pair. Both male patients (BMI: 31 kg/m^2^ and 29 kg/m^2^) underwent a PET-scan for staging of melanoma. In both cases, lymph node metastases were detected in the left axilla (red arrow). Axial PET-images of the axilla (C/E) and the liver (D/F) show both the tumor and background uptake. Images with a black frame indicate the use of long-axial field-of-view (LAFOV) PET in UHS mode for 360 s acquisition time (A/C/D) and with a grey frame simulated short-axial field-of-view (sSAFOV) PET with HS mode for 120 s acquisition time (B/E/F). Scale bars indicate the SUV-window used for both patients.
